# Managing the unknown or the art of preventing SARS-CoV-2 infection in workplaces in a context of evolving science, precarious employment, and communication barriers. A qualitative situational analysis in Quebec and Ontario

**DOI:** 10.3389/fpubh.2023.1268996

**Published:** 2024-01-08

**Authors:** Daniel Côté, Ellen MacEachen, Ai-Thuy Huynh, Amelia León, Marie Laberge, Samantha Meyer, Shannon Majowicz, Joyceline Amoako, Yamin Jahangir, Jessica Dubé

**Affiliations:** ^1^Institut de recherche Robert-Sauvé en santé et en sécurité du travail, Montréal, QC, Canada; ^2^School of Public Health Sciences, University of Waterloo, Waterloo, ON, Canada; ^3^School of Rehabilitation, Université de Montréal, Montréal, QC, Canada

**Keywords:** COVID-19, frontline workers, communication process, workplace, health information, occupational health, information—access and interaction, qualitative study

## Abstract

**Introduction:**

The issue of communications in the public space, and in particular, in the workplace, became critical in the early stages of the SARS-CoV-2 pandemic and was exacerbated by the stress of the drastic transformation of the organization of work, the speed with which new information was being made available, and the constant fear of being infected or developing a more severe or even fatal form of the disease. Although effective communication is the key to fighting a pandemic, some business sectors were more vulnerable and affected than others, and the individuals in particular socio-demographic and economic categories were proportionately more affected by the number of infections and hospitalizations, and by the number of deaths. Therefore, the aim of this article is to present data related to issues faced by essential workers interacting with the public and their employers to mitigate the contagion of SARS-CoV-2 (COVID-19) at work.

**Methods:**

Following the constructivist paradigm, an interpretative qualitative design was used to conduct one-on-one interviews with precarious/low-wage, public-contact workers (*N* = 40), managers (*N* = 16), and key informants (*N* = 16) on topics related to their work environments in the context of COVID-19 prevention.

**Results:**

This article has highlighted some aspects of communication in the workplace essential to preventing COVID-19 outbreaks (e.g., access to information in a context of fast-changing instructions, language proficiency, transparency and confidentiality in the workplace, access to clear guidelines). The impact of poor pre-pandemic working relations on crisis management in the workplace also emerged.

**Discussion:**

This study reminds us of the need to develop targeted, tailored messages that, while not providing all the answers, maintain dialog and transparency in workplaces.

## 1 Introduction

The issue of communications in the public space, and in particular, in the workplace, became critical in the early stages of the SARS-CoV-2 pandemic. This issue was exacerbated by the stress of the drastic transformation of the organization of work, the speed with which new information was being made available, and the constant fear of being infected or developing a more severe or even fatal form of the disease. Some researchers in the fields of communication in workplaces and knowledge transfer suggest that it is enough to be open and transparent, not to withhold information, and to put in place good communication practices and strategies (1, 2). Irrespective of the form of communication, the right interpretation depends on the definition of the “object/subject” to agree upon, the intent behind the message to share, and the communication style used (3, 4). Most theories on communication would concur that the effectiveness of the message depends at least in part on a common definition of the “object” to be discussed (5), whether at the level of mass communication (6), organization (7) or interpersonal interactions (8). This is the first condition for dialog and exchange. In other words, we need to agree on the definition of the object/subject of the communication prior to forming our opinion on it: the choice of words, understanding of their undertones, and different levels of possible meanings, perceptions, and understandings, as well as the level at which the current exchange takes place and which level and tone should be promoted to enable people to make sense of and adhere to the message they receive (9). For instance, if talking about viruses, we need to know what the word “virus” refers to in its primary, microbiological sense, and possibly, metaphorically, in a figurative sense, e.g., designating a threat, a wound, a danger related to an ideology, a group of individuals, a fad, or a trend that is deemed pernicious or undesirable. The recent introduction of the term “infodemics” (i.e., inaccurate, false, misleading, or unproven information) into the world of humanities, social sciences, and public health research is a good example of this phenomenon, which directly affects the management of the COVID-19 pandemic (10–12). This is particularly evident when the COVID-19-related health policy-making process and the science-making process intersect, leaving room for many unknowns and possible contradictions and generating public feelings of uncertainty and confusion, and possibly of mis−/distrust (13, 14).

As early as January 2020, the WHO alerted governments around the world of the SARS-CoV-2 (so-called 2019-nCov) outbreaks in the city of Wuhan, China, and its alarming contagiousness (15). When a pandemic was formally declared in March 2020, the main affected countries began taking drastic measures to control the situation. Workplaces have not been exempt from having to implement seemingly inconsistent measures to protect their employees (16). Eliminating or controlling the potential source of a SARS-CoV-2 infection risk, as prescribed by many national occupational health and safety (OHS) laws, is not a straightforward process, especially when the scientific community does not agree upon the virus’ various modes of transmission in a closed environment, as in the case of aerosol transmission (17, 18). Is the wearing of surgical masks sufficient or should N95 masks be recommended? In the early stages of the pandemic, workers requested accurate and fair information, but their employer or even their trade union often had to deal with unclear government guidance on certain issues when, for example, scientific advice differed from one organization to another.

Public health and OHS authorities have worked—sometimes jointly, sometimes in parallel, depending on the different national governance structures—to provide the public with practice guidelines, fact sheets, and procedures to follow for any infection or outbreak (11). Although effective communication is the key to fighting a pandemic (19), some economic activity sectors were more vulnerable and affected than others, and some socio-demographic categories were proportionately more affected by the number of infections and hospitalizations, and by the number of deaths (20, 21). Poor living conditions and various social or economic vulnerabilities (e.g., housing, transport, access to communication means, access to healthcare facilities, language and cultural barriers, working environment, migratory status) amplified workplace health and safety issues (22–26).

Such epidemiological differences between groups, especially minority ethnic groups are amplified by the prevalence of other public health problems (e.g., air pollution, malnutrition, population density) that reveal not only disparities, but also social inequalities in health (21, 27–29). Very early on in the development of the pandemic, it became clear that public health and OHS needed to be better integrated or harmonized, necessitating more efficient communication between institutional bodies and in their strategies for relations with the general public (11).

The increased vulnerability of certain categories of workers to the risk of occupational injury has long been known, although an effective institutional response has been slow to emerge (30). Many of the so-called essential workers during the pandemic find themselves in vulnerable and precarious situations. They include precarious or low-wage workers, agency or limited-contract workers, under−/unprotected workers, ethnic or racial minority workers, immigrants and workers with poor language skills, and ageing, low-educated, or disabled workers (16, 23, 31). The gendered nature of precarious employment has also long been known (32, 33) as men and women are not equally represented in the various industries, and the pandemic has not affected them in the same way (31). Not all of these workers have equal access to information and appropriate job training (and occupational risk) (30, 34), and as such, communication efforts (or lack thereof) have left them at increased risk.

This article reports some results from a broader qualitative study whose main objective was to explore in a comprehensive manner how essential workers interacting with the public and their supervisors understand the situation, make choices, and navigate through public health recommendations to mitigate the contagion of COVID-19 at work.^1^ Many essential workers were employed in jobs involving direct contact with the public, many of them in various forms of precarious work.

In this study, precarious work has been defined on the basis of the following dimensions of income and revenue (i.e., low wage, platform workers), job security and type of employment (i.e., temporary placement, agency work), and the enforcement of rights and protection (i.e., social benefits, paid sick leave) (34–36).

The aim of this manuscript is to present data related to issues workers face in accessing medical or public health information and accessing clear and sound guidelines. These data highlight the challenges associated with rapid changes in public health guidelines or instructions and their impact on the communication and information management and transmission chain.

This study received ethics approval from the University of Waterloo Human Research Ethics Board (Protocol certificate number: 42449). Informed participant consent was obtained verbally and recorded before a telephone or videoconference interview.

## 2 Methods

Following the constructivist paradigm, which posits the existence of multiple social realities constructed from individuals’ perceptions that vary over time and context (37), an interpretative qualitative design was used to address the objectives of this study.

The constructivist paradigm is a philosophical and theoretical framework which asserts that reality is socially constructed and subjective, shaped by individual experiences, interpersonal interactions, and interpretations. In the context of research, the constructivist paradigm can have a substantial impact on methodological choices, data interpretation, and the overall design of a study. It prioritizes qualitative methods and in-depth exploration of how people make sense of their everyday world and the influences on their choices and reasoning. Data interpretation involves recognizing and understanding multiple perspectives and the overall design of the study is characterized by flexibility, iteration, and participant involvement. This means that rather than imposing pre-defined categories or theoretical frameworks on the data, constructivist researchers often allow themes to emerge organically from the data. Researcher reflexivity is also a key element of constructivist research, recognizing the impact of the researcher on the study.

Workers, managers, and key informants were recruited using purposive sampling strategies, combined with elements of snowball sampling. The inclusion criteria for workers were: (a) over 18 years old; (b) working in an essential sector (i.e., essential to preserving life, health, and basic social functioning) during the first-wave SARS-CoV-2 lockdown; (c) low-wage workers (approximately CAN$4 above the provincial minimum wage); and (d) working in a public-contact job (i.e., having physical proximity with clients in order to deliver the service). These criteria were established to focus on the experiences of workers who were already in a precarious situation when the pandemic began and had to maintain work deemed essential. The vulnerability of these workers in terms of OHS and protection is well documented by research; the aim here was to see how the pandemic might affect their already precarious working conditions and how this precariousness might influence their choices in terms of risk prevention and control. Inclusion criteria for managers and key informants were: (a) over 18 years old; (b) holding a management or supervisory position in an essential sector that hires precarious workers or an organization dedicated to the defense of workers’ rights or the promotion of OHS (e.g., OHS prevention and inspection, legal clinics, advocacy NGOs, trade unions, public health). Interviews were held from August 2020 to March 2021. Since interviews in Quebec started and ended later than in Ontario for logistical reasons, researchers on the Quebec team added questions about the second wave (September–December 2020) and the vaccination campaign (begun in mid-December in both provinces). Seventy-two participants were selected and divided into three groups: low-income workers (*N* = 40), supervisors/managers (*N* = 16), and key informants (*N* = 16). Two participants (trade union representatives) were interviewed together at their own request (called “paired-depth interviewing”: people from the same organization but holding different titles and hierarchical positions; 38). Semi-structured interviews were held in English (*N* = 36), French (*N* = 33), or Spanish (*N* = 3), and conducted according to an interview schedule on topics related to their work environments in the context of COVID-19 prevention (see Table 1). The interviews allowed sufficient time for participants to raise any other issue or theme they considered relevant to our understanding.

**Table 1:** Guide for interviews with workers, managers, and key informants.

Workers	Description of their work responsibilities and working conditions The health measures implemented in their workplaces Their risks of COVID-19 exposure and transmission in the work context Changes to be made to better protect workers Their decision-making process about taking time off to go for COVID testing or when symptoms are present Discrimination The second wave (QC only) Anticipated view of the vaccination (QC only)
Managers or key informants	Main health risks for their employees Management of COVID-19 in the workplace Challenges of workers’ returning to work after a COVID-19 absence Frequency of leave requests Changes needed to better protect essential service workers Issues faced by low-wage public-contact workers

Interviews were recorded and transcribed by a professional transcriber using a word-processing software, and then transferred to qualitative analysis support software NVivo for researcher coding, inference, and interpretation following baseline qualitative content analysis (39). Data analysis was based on situational analysis (SA) (40), using conceptual mapping to frame and analyze the workplace situations and social worlds. The analytic process was iterative, involving constant weekly team meetings to discuss emerging themes, situations, and possible logical relations and hypotheses.

The majority of workers were women (65%), and despite some missing information on origin, roughly equal numbers were Canadian-born or immigrants. About 33% were union members (mostly in Quebec), more than half (25/40) had a college or university degree, and about 18% worked at two or more jobs to supplement their income. Half of the workers reported being part of a racialized group. The average age was around 37 years. A majority of workers were employed in the retail sector (*N* = 19), health and social services (*N* = 8), and accommodation and food services (*N* = 4), and the others, in education, security agencies, agriculture, manufacturing, hairdressing and beauty, and transport. All workers but one were in direct contact with the public. To preserve the anonymity of our participants, we have used pseudonyms in the extracts presented in the results section.

Thematic development is a crucial aspect of qualitative research, particularly in the context of grounded theory methods, including situational analysis. It involves the systematic identification, analysis, and refinement of themes or patterns within the data collected. Researchers began to categorize and label data from an initial set of codes, often short descriptive labels attached to segments of data (e.g., working conditions, pressures at work, what is risky, plans vs. practice, customer problems, response to risk measures, sick leave-COVID, sick leave-any situation, policies, organizational changes needed, personal or home issues). New data were compared with existing codes and categories to identify similarities and differences. This iterative process helped the researchers to refine and expend their codes, gradually building a more nuanced understanding of the data. The initial codes did not include communication issues. This issue arose during axial coding when the researchers explored relationships between different codes. This involves linking categories and subcategories to reveal patterns and relationships. Axial coding helps to identify central themes and concepts that emerge from the data. The themes of communication are described below using quotes from the interviews. From these quotes, the researchers attempted to make connections between categories of meaning and, through discussion between team members, to produce the conceptual maps presented at the end of the Results section.

## 3 Results

The data analysis identified several themes (to be discussed in other articles). The theme discussed in this article is communication in the workplace in the context of a COVID-19 health crisis.

**Figure 1:**
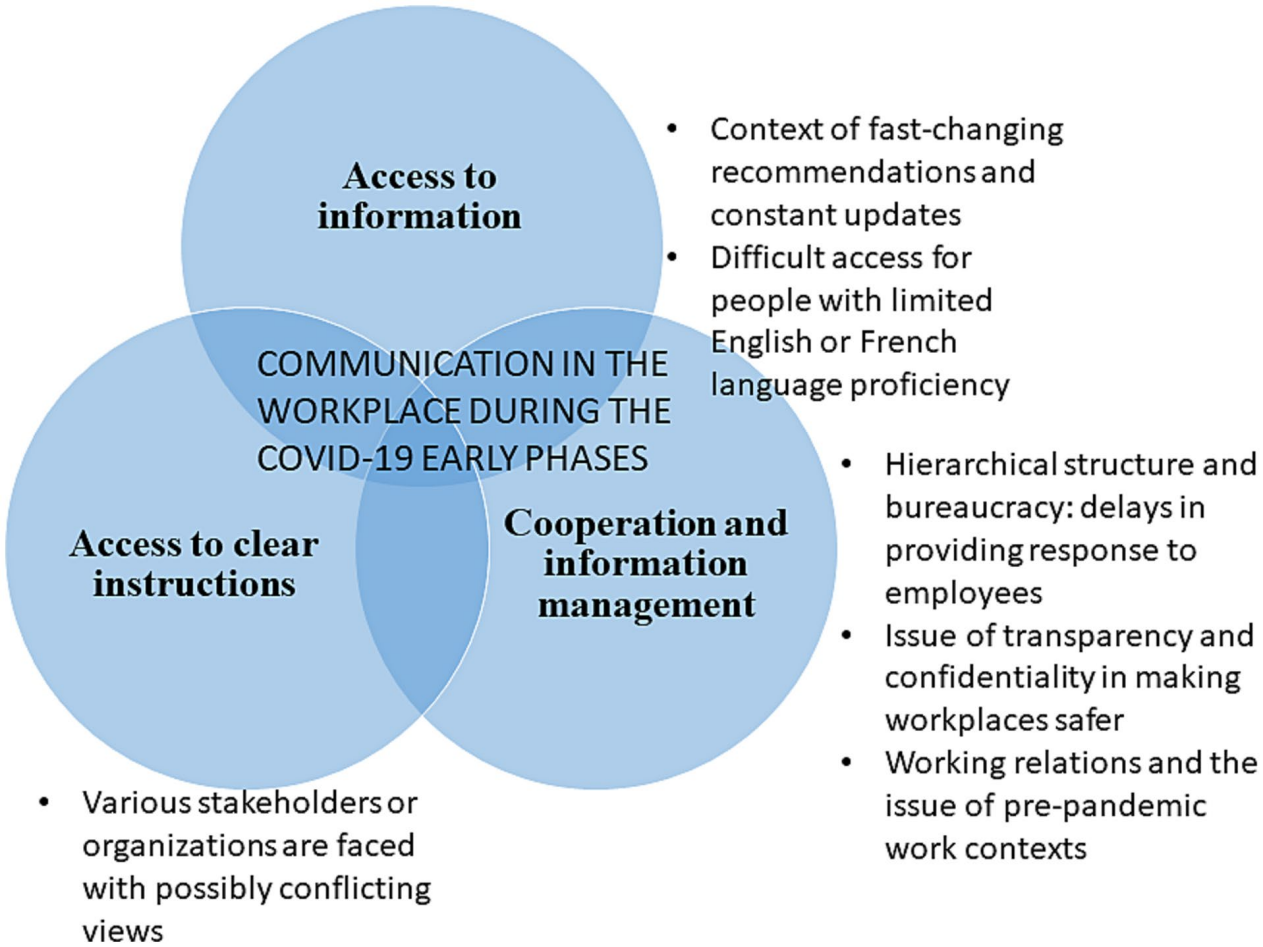
Subtopics of workplace communication during the pandemic.

In Figure 1, communication issues in the workplace were divided into three subtopics. The first subtopic was access to information in a context of fast-changing recommendations and information updates. The notion of access is also expressed in another way, where the earliest information might be accessible in terms of location (addressing the ‘where to find it’ question), but hard to understand due to French or English language-proficiency issues in both provinces. The second subtopic was access to clear guidelines when several organizations are involved and must coordinate their actions in the field. The third subtopic pertained to cooperation and information management among complex hierarchical structures and bureaucracies, creating delays in the provision of clear guidelines, indications, or timely response to employees (see Figure 1).

### 3.1 Access to information

#### 3.1.1 Context of fast-changing recommendations and information updates

The SARS-CoV-2 pandemic was a new phenomenon requiring everyone to adapt quickly and implement protective measures in accordance with public health instructions. However, they were no better prepared to fight the virus, as it was still largely unknown by scientists (or epidemiologists) only a few months earlier. Thus, sometimes the institutional response gave the impression that the State did not provide clear enough guidelines or that they changed too quickly, as the following extracts illustrate:

…in addition to not knowing, in addition to changing often, when we have the instruction, we do not know how it applies until a week later. So you’ll understand that yes, yes, we have the instruction, [but] how does it apply? We do not know, and we cannot tell you. In the meantime, well, everyone does whatever they want for a week, and then a week later, we realize that no one has done it properly, and so it changes again. (...) The problems were continuous, you know, happening every day, and the ministerial orders changed daily (Véronique – key informant, president of a local trade union).

For this worker in the social services sector, who works specifically with homeless people, the expectation of clear instructions was palpable:

Well, I read the information, but it keeps changing, the screening clinics are no longer there the week after, but what do you do, you know? I think that people should be told that there is a lot of information, and that it keeps changing. At one point it was the mask, the faceshield, and then that changed, then came the Plexiglas, you know, I try to follow the instructions, but (...) basically, they should say clearly what you should do (Catherine – worker, community-based organization working with the homeless).

Public health and OHS authorities worked collaboratively to develop fact sheets, and sometimes with the help of local NGOs dedicated to specific populations such as newcomers or cultural and linguistic minority workers. Sectoral associations and professional bodies also developed their own material. Fact sheets and information tools were created for specific workplaces or sectors. However, these efforts did not reach or hardly reached sectors such as food delivery workers, who were not deemed a priority according to some jurisdictions.

During the pandemic, we saw a lot of people who stopped working [in the workplace] and found the option of working on or using platforms, right? For food delivery, etc. (...) one day, when I was looking at the information from the CNESST [Workers’ Compensation Board] in the different sectors, at the guides they had developed, there was nothing for these sectors (the gig, platform economy) (...) there were people, from (*name of platform*) who had caught COVID. Because they were delivering food. There were no specific instructions for them (this sector, this specific task) to protect themselves against that (Alejandro – key informant, volunteer for an NGO dedicated to immigrants).

While the rapid change in prevention measures is a concern for many workers, it is also an issue when a worker who has been absent for a period of time returns to work. The challenge for the employer is to ensure that the information on the latest implemented health measures has been communicated and that the employee understands it. Here is what this prevention-inspection agent told us:

But going back to work, the same thing happens again. While I was away, what’s changed, what’s new? What are the... what’s ‘a reminder of the rules’? It really depends on the work environment. In a restaurant, it’s simpler, but in a daycare centre, it’s something else. A hospital, a hospital environment, a [long-term care home], that’s another thing. You know, in all cases, when someone returns to work, the worker must have the information he needs, so the employer who gives him the information must make sure that he really understands the instructions (Mathieu – inspector, Workers’ Compensation Board).

While the expectation for clear guidance was palpable among many participants, it also suggested that the changing nature of the information could have been made more explicit to better prepare people to receive information that is bound to change rapidly as knowledge evolves (rendering obsolete what was assumed true a week earlier). Transparency on the part of health authorities and the government about the limited knowledge about COVID-19 might have better prepared the public to receive information that was subject to rapid change.

#### 3.1.2 Context of difficult or limited access for people with limited French or English language proficiency

Communication and access to information in a language which workers know and in which they are sufficiently fluent is another important issue that emerged from the data collected in this study. In times of crisis, ensuring access in a language that is understood by everyone to ensure that they all understand the instructions seems logical or common sense. Yet language barriers were reported, as this nurse indicates:

And so, you know, identifying that language barrier is number one for me because I do not want to continue with the case investigation if I know that they are not understanding me, right, and they are not getting that information so I would either transfer the case to someone who can communicate with them or that (…), so language is one big thing (Piper – key informant, public health nurse).

Some categories of workers, such as temporary foreign workers in Canada, are more vulnerable, as no language-based selection criteria (proficiency in either official language) apply to them (as for economic immigrants) even though they are exposed to many sources of workplace hazards without access to adapted or translated instructions, notices, or sectoral information sheets.

Well, it’s a bit complicated because there are several of us and we come from different places, and there are people who come from rural areas who are not so used to being in places where there are a lot of people and there are even (*sic*) people who cannot express themselves very well in French. This makes us shy at the same time and we cannot talk to them because they speak French, and there are very few who speak Spanish (Miguel – foreign farm worker, translated from Spanish).

The possibility of obtaining information, and the existence of such information, is probably what first comes to mind when we think of access to information. However, access to information is also about the comprehensibility of the transmitted message. From an anthropological point of view, most of the world’s languages contain different levels of complexity and sophistication which may be less “accessible” to some people depending on their level of education or level of knowledge of a specific language (e.g., specialized language, jargon specific to a given profession or sector, or even regional or class-based patois). This discrepancy can alter the understanding of the message, or even compromise it by suggesting an interpretation that is not the one originally expected by the sender, as this director of a public health unit suggests:

Typically, these manufacturing settings are, you know, are conducive, I would say, to transmission, simply because of the fact that you have, in many cases, you know, low-wage workers who, you know, to some degree, may not have the education from an IPAC—infection, prevention and control perspective—so that’s certainly one of the limitations. Education, in general, may be a limiting factor, as well, as it relates to this population (Brian – key informant, director, public health unit).

According to this public health nurse, effective communication needs to be attentive to education and health literacy:

And then even without language, even if they can speak English fluently, but they are not health care, they are not in the health care field, they are not in the public health realm. And so for them, when you say things like period of communicability [of the virus] or when you say acquisition or transmission exposures, it can get very confusing, and so what I try to do is obviously just kind of break it down into really simple terms, layman’s terms, pretty much. Just pretty much say like, ‘OK, where did you go? Where could you have potentially caught it?’ And then going over [the term] isolation, just saying, you know, do not worry about the term isolation (Piper – key informant, public health nurse).

This public health nurse noted the importance of clear communication in terms of what language is best understood by the contact (calling in a multilingual colleague as needed). Terminology and avoiding jargon are important as well. Other issues prior to COVID-19 transmission, which may seem more trivial or self-evident, also represent a communication challenge, such as the wearing of masks. Therefore, obtaining the latest information on the best protective measures and protocols may well be an issue when even how to use procedural medical masks is difficult to convey (although such information has been known for a long time). As this employee of a large supermarket pointed out:

…but we are not, after we take our masks off, we are not washing our hands before we touch our face; we are washing our hands, we are taking our masks off and then we are touching our faces, and she said that, that has been a, a great source of misinformation towards the public, you know, because whatever bacteria or anything that’s accumulated on the outside of the mask is now on our hands and now in our eyes, and now in our noses, and now in our mouths, you know? (Claire, worker, multinational retail corporation).

This accessibility issue not only concerns the knowledge of official languages or the existence of multilingual material, but also a relationship that can sometimes be distant or strained between health organizations and certain sections of the immigrant or cultural minority population.

…it’s useful to speak the language spoken by the person when it is not among the official languages; it creates a bond of trust (...) You know, I do not come here only to, let us say, just as a public health representative, I also come because you are a citizen and you are a human being (...) You know, it shows a certain interest, deeper than just coming as the public authority (Roxane – key informant, public health practitioner).

Language is a means of creating bonds, of breaking the chains of mistrust and misunderstanding, well beyond its instrumentalization for the purpose of transmitting messages of public interest.

### 3.2 Access to clear guidelines when several organizations (health institutions, ministries, associations) are involved

Clarity of information is another issue that emerged from our data collection and subsequent analysis. In this section, the issue of the presence of several stakeholders or government agencies involved in the development and implementation of health protocols is discussed. Depending on the availability or accessibility of materials dedicated to specific sectors, the impact of these issues may have varied. Our data is limited on this subject, but the experience of the following taxi company manager and school bus driver, both in the transportation sector, nevertheless raises some questions:

The thing I would say, and that is not obvious, is that between the parties involved in setting up protocols, nobody talks to each other. That’s, that’s rough, you know, take Public Health (...), the INSPQ [public health institute] (...), the Ministry of Transport (...), the Employers’ Council (...), where everybody says something different, [and] the SAAQ [public automobile insurance plan]. Everyone has a different opinion or makes a different recommendation, [so] for us it’s extremely complicated (...) You know, I had to call the elected officials. I had to call the Ministry. I had to call doctors from Public Health to get the right information, and each time I got different information; I had to make, I had to amalgamate this information and sort it out myself and put in place what was logical to me. But, you know, I told myself, that’s just the way is it (Camille – manager, taxi company).

And:

I think that perhaps the Ministry of Education should not have interfered; that’s what this is about. [They should have] let the authorities—the INSPQ [public health institute], the [Workers’ Compensation Board], all the public health authorities—dictate what was appropriate and what wasn’t. Unfortunately, we have ministries that sometimes interfere when they should not (John – worker, school bus driver).

This overload of information can therefore cause confusion among workers as well as the general population, and possibly frustration at finally having to synthesize the information themselves according to what ‘logic’ tells them. Similarly, the inconsistency between the different public health bodies affects the credibility of these organizations and the level of public support. Yet referring to a credible and knowledgeable interlocutor is the first thing that every employee, supervisor, manager, or stakeholder would like to do. This suggests that people wanted a message; they were motivated to try and find the message (i.e., not resistant to it). In other words, a typical barrier of an unreceptive audience was perhaps not at play, but rather, the message was hard to find despite their best efforts and readiness to act.

Lack of coordination between various government entities often leads to delays in information dissemination in the service sector. For this union leader in the education sector, one of the main challenges was the delay in the information transmission chain in a context of rapidly changing safety instructions.

So the school had to, the school board had to put things in place [e.g. whether or not masks should be worn, what to disinfect and frequency of disinfection, addition of new tasks] without having the right instructions, without being aware of them because they were too last minute, so it was done so much, like fast, on the fly, but that continued to increase people’s anxiety (Karine – key informant, local trade union).

Here again, the rapid change in relevant information on financial compensation schemes for employees, their union representatives, and employers too was a source of considerable confusion and uncertainty, as people wanted answers to their questions and, more importantly, did not expect answers to be so transient.

There is a major flaw, and that is the lack of understanding of the countless measures that were put in place for workers (...) They introduced a host of measures that varied over time, in both amount and eligibility rules, so that this created a certain amount of confusion among workers and, and the entire work environment, both union and management and employers’ lawyers. Things were always somewhat vague. There was never any certainty and we had to give answers to these people by saying ‘here’s how it is now, but it could be something else next week’ (Patrick – key informant, local trade union).

This same union leader argued for a more uniform or centralized system of information management, which would have created a stronger sense of safety, despite the changing instructions:

I think it would have been better to (...) I think we would have benefited from having a single system that would have done everything, where we would have said ‘this system, we guarantee that this will be the case until such and such a time.’ So for the worker, there would have been a certain, a certain feeling of safety (Patrick – key informant, local trade union).

There may also have been a combination of factors, for example, the effect of rapid changes in safety and prevention instructions regarding COVID-19 and the so-called “bureaucratic structure” of the institutions under the Ministry of Health, which presumably led to undue delays between requests for information, the receipt and processing of requests, and institutional responses. This was expressed by a human resources department director in a health care facility:

The instructions changed extremely quickly so that, you know, an employee who’s a bit afraid and who does not really know [what to do], and then we say ‘put on your glasses,’ ‘take them off, you do not need them.’ But you know, you have to explain to him why we are doing this, what’s the point. And you know, when it comes from the Ministry, well, sometimes we, we make up the meaning (laughs). You know, yes, we can question the Ministry but sometimes it takes three weeks before they answer us (...) You know, there’s, there’s a bureaucracy that’s not easy, especially since we have the CIUSSSs [integrated university health and social service centres]. It’s good to have centralized certain things for more consistency, but for other things, this means [additional] delays. And in a health crisis, there cannot be any delays (Constance – human resources department director, CIUSSS).

There are certain contextual elements to be clarified in this excerpt, as this participant is referring to the merger of institutions that took place a few years ago in Quebec, during the reorganization of health and social services institutions. This reform, carried out in the name of effective public management and more effective patient care, led on the one hand to more centralized management of staff and programs, which this informant felt was more coherent, but also led to more bureaucracy in the information transmission chain. Expectations of quick, clear answers or instructions are hard enough for authorities to meet in normal times, but are that much harder to meet during an unprecedented health crisis, especially in this sector that has experienced an extremely high number of outbreaks and deaths given its very high-risk clientele.

### 3.3 Information management

#### 3.3.1 Communication in the workplace, transparency, confidentiality, and the issue of making workplaces safer

While access to information posed a significant challenge for workplaces during the pandemic, particularly in the early stages, in terms of how to obtain information and its comprehensibility (level of language, multilingual tools, etc.), it also posed an internal challenge for companies concerned with protecting their workers while ensuring the confidentiality of personal health information. What information should be disclosed for the sake of transparency and worker protection, and how it should be disclosed to ensure maximum discretion without compromising the flow of information needed to develop the best prevention measures? This pharmacy manager expresses these concerns as follows:

In addition, it [the information we receive] tells us that everything is confidential. So we do not know if people are coming to work or not. For example, we had one person who was laid off because she was in a high-risk age group. But after that she left [for good]. But we never knew if it was because of her age, because she had symptoms (of COVID-19), or because she’d been tested (and was waiting for the results). At the lab, it’s the same. In fact, we are always told that people are on holiday. But in reality, you do not know if they are on holiday or if they have symptoms (Sofia – supervisor in a pharmacy).

While respecting employees’ right to confidentiality, a minimum of transparency on the part of the employer regarding his own actions and strategies seems to be required, as expressed by this Workers’ Compensation Board inspector:

Often, workers would leave work from one day to the next. People would learn that one of their coworkers had taken a COVID test. They learned that the test was positive. Some employers are transparent, others a little less so. A lot of rumours were going around. I heard that a fellow in human resources was removed for COVID, so what does the employer do? We do not know. Are any people infected, any actions to be taken? We do not know what it is (...) I felt that there was a lot of panic among the workers at that time, especially between March and May (...). People want, they just want to be reassured. They just want to know what’s going on, to know what the employer has done (Mathieu – inspector, Workers’ Compensation Board).

This suggests a degree of transparency, despite the paucity and lack of certainty of the information to be shared with employees. It allows for a certain degree of openness and frankness. However, there are arguably conditions that must be met to achieve this, as discussed in the next section.

#### 3.3.2 Working relations and the issue of pre-pandemic work context

Transparency has a blind spot: the prevailing climate and working relationships within the workplace at any given time and which may hinder smooth communication. As this union leader points out, poor working relations before the pandemic would only worsen the conditions for communication during a health crisis:

I do not know if it’s limited to work relations (...) but where work relations were [already] bad, that’s where they were the worst during the pandemic. So when communications were not going well before, it became hellish, hellish (Véronique – president, local trade union).

In terms of counter-examples, we have compiled some data that reflect the positive side of communication in a healthier work environment, one that is more conducive to solidarity between workers and even a certain proximity between managers and staff. As one supermarket branch manager put it:

So I was recognized as a manager who was close to the customers, close to the operations, supporting the team. So without necessarily working physically with [my] coworkers, I was, I was very, very, very close to them physically (Ian – manager, grocery store).

Here is how a coordinator of a homeless shelter also talks about it:

I talk to them regularly, I talk to everyone about twice a week, so that we... I try to see how they are doing, how their motivation is, what’s going on in their daily work activities, [or] if they have any problems with their work (Gaston – homeless shelter program coordinator).

In unionized environments, the atmosphere can be one of cooperation and solution finding:

They worked together. You know, the union would come in and say, ‘Oh no, this is not going well.’ And she (last name of the executive director of the school service centre) would say, ‘Oh, okay, we had not thought of that. What if we did that [instead]?’ You know, they used to work like that here; yes, we lost people in the battle due to fatigue and overload, but many fewer than elsewhere. She [the director] found solutions. They worked together (Karine – president of a local trade union).

**Figure 2:**
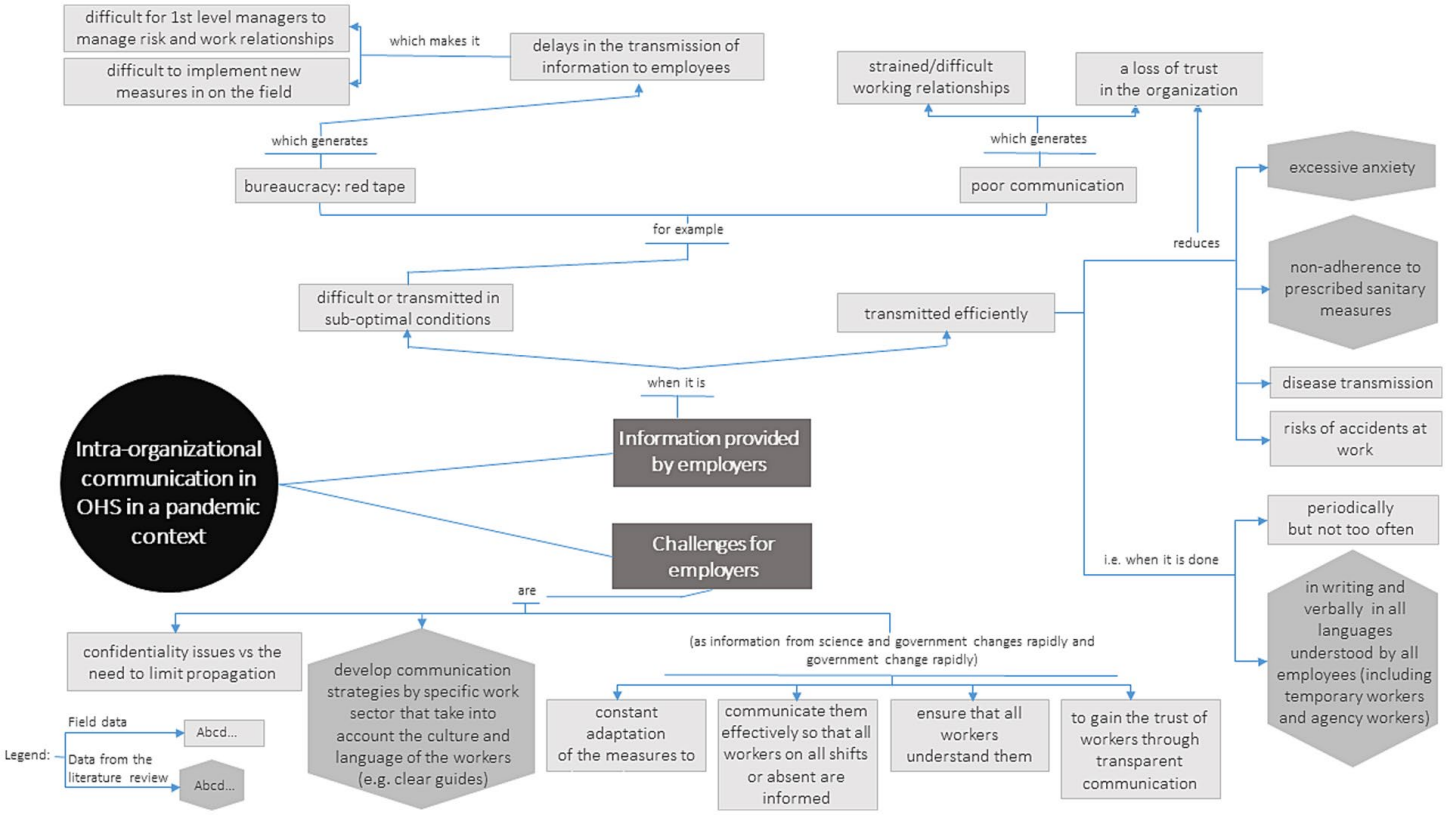
Intra-organizational OHS communication in a pandemic context.

**Figure 3:**
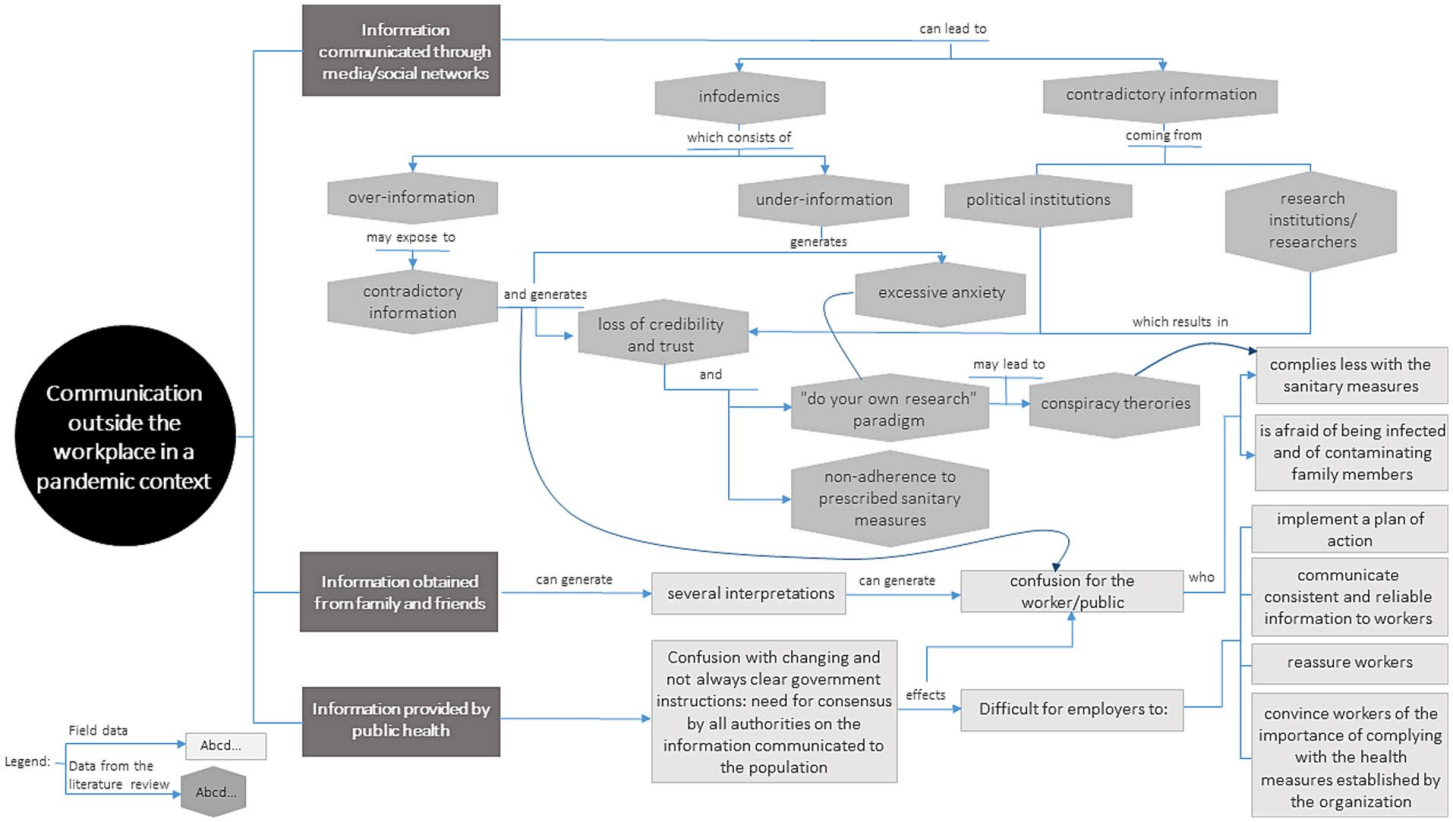
Communication outside the workplace.

To summarize and illustrate the salient concepts regarding communication in the COVID-19 context, as well as their logical interconnections, Figures 2, 3 present conceptual maps on intra-organizational OHS communication in a pandemic context and communication in the COVID-19 context outside the workplace, respectively. Although the subject of this article focusses specifically on communication within the workplace, we have chosen to illustrate aspects of communication outside the workplace also, as this communication directly impacts both workers and organizations. Based on the data collected, the health crisis has shown that the boundaries between mass communication, general public communication, and communication within organizations are clearly very thin and porous. Furthermore, not only have we included concepts from the field data in these conceptual maps, but also those from a previous scoping review (23), informally supplemented with keywords related to COVID-19 risk communication in the workplace. This provides a more holistic understanding of the concepts/knowledge related to this theme.

## 4 Discussion

The new coronavirus spread very rapidly from when it was first identified, taking all the world’s governments by surprise. By the time the WHO formally declared a pandemic in mid-March 2020, very few states had a well-developed plan of action, and they had to act extremely quickly with drastic and unprecedented measures: lockdowns, closing of non-essential businesses, etc., which had devastating effects on the economy, employment, and the ability of some companies to continue operating. Frontline workers in essential businesses were vulnerable during the early phase of COVID-19. They were often low-wage and precarious workers (16) or facing communication issues involving lack of proficiency in their host country language (41). Like governments, workplaces in turn had to react quickly and support their employees.

Our data suggest different variations on the theme of workplace communication during the earliest stages of the pandemic. Access to information in a context of fast-changing recommendations or instructions was one such variation, along with access for people with limited English or French language proficiency (or any other language serving as a working language and used in official written or verbal communication) and access to clear guidelines when various stakeholders were involved and sometimes had conflicting views or instructions, and deficient cooperation and information management. The nature of pre-pandemic working relations and communication patterns was also reported as having tremendous impact on management’s ability to provide satisfactory responses during the pandemic crisis.

In our study, the difficulties of accessing and understanding the right information were reported by many participants, whether ordinary employees, managers, or stakeholders. There was an element of urgency, as they had experienced difficulties accessing information quickly, which in turn hampered their ability to implement appropriate actions. In the world of work, which is our main focus here, we found that the sources of information were also diversified, and that the same uncertainties and confusion could be present in different workplaces: information transmitted by employers, conveyed through the internet and social media, or transmitted by public health and OHS authorities (and not necessarily in sync), in addition to information shared during daily exchanges between people in the immediate environment (neighborhood, family, social network). All of this can be a source of confusion that often leads people to draw their own interpretations (42, 43). In the world of OHS and disability management, the uncoordinated presence and sometimes concurrent actions of several health specialists and experts can lead to this kind of confusion through differential diagnoses, and this has been shown to significantly alter the therapist-patient relationship and, not least, trust in the system (44, 45).

This empirical study, combined with the results of other studies, clearly indicates that under-information may be an obstacle when science is being developed at the same time as different communication strategies are emerging in the workplace and in public health. However, over-information is no less damaging, and this calls for a more sustained drive for coordination, concertation, and the introduction of a rapid and updated, single source of the latest knowledge in order to deliver a coherent and consistent message to the public, including workplaces (42).

In a world where mass communication and social media have become pervasive, it is not always easy to separate the relevant information from the irrelevant. Moreover, not all individuals are familiar with or instinctively consult a public health or OHS agency website for answers to their questions. Moreover, it has been reported elsewhere in numerous academic works that precarious workers or workers in vulnerable situations are unaware of or have little knowledge of their OHS rights and protections, and indeed, sometimes they have only a vague idea of even the existence of a workers’ compensation board (referred to as a WCB in OHS literature) in their respective jurisdiction (46–49). In Montreal, early in the pandemic, working groups were formed and met regularly to address issues related to immigrants and precarious workers, COVID prevention, and OHS. It was reported that “ethnic” or multicultural media that may use languages other than the official ones in the country concerned (newspapers, radio, etc.) had been under-utilized to disseminate information about the pandemic (50). Fact sheets were prepared in multiple languages to convey information about the disease, workers’ rights at work, financial assistance, isolation instructions, face coverings, and recommendations about grocery shopping and working at home (51). In Quebec and Ontario, standard guides and specific sector-based toolkits for workplaces were produced by OHS authorities (WCB) in both French and English, with Spanish instructions for the Agriculture sector, about a third of whose workforce is foreign temporary workers, almost exclusively from Latin America (52, 53). Despite the efforts made, their access to this information was uncertain, for how could they access it if they were unaware that these bodies even existed. This raises the issue of how to establish more robust communication platforms to disseminate public health/OHS information, strategies, or models, particularly for workers with precarious employment and/or marginalized public-contact workers from diverse cultural backgrounds.

Lack of access to information, partial access to information, or ambiguous information can lead to information seeking from unexpected or less-than-ideal sources (e.g., fake news, conspiracy theories, outdated information) (11). As the saying goes, nature abhors a vacuum, and the uncertainty created by this apparent vacuum can drive people to other sources of information (12). Inadequate communication strategies can lead to ambiguity and confusion (54), and do not help build trust between health authorities and the public (42, 55, 56). It is therefore likely that these inconsistencies will increase public anxiety and undermine the credibility of the science and, consequently, the adherence of workers and the public to health measures or restrictions. Transparency and consistency of messages is important, even if the information is subject to change (42, 57). Transparency implies a certain management style, which, in turn, is based on trust. When relationships are strained, i.e., not conducive to exchange, and when interpersonal and organizational relationships are fragile, disrupted, or broken, it becomes difficult to think about transparency and openness. It may seem natural to see attitudes of withdrawal and silence appear. This can become a vicious circle, with mistrust or tension feeding opacity, opacity feeding mistrust, and so forth. Current knowledge does not allow us to state whether this is more prevalent in contexts of precarious work (e.g., job insecurity, piecework income, no social benefits, no long-term contract, and temporary status) and where there is a history of labor disputes and litigation.

In addition to issues related to the absence of information, its lack of accessibility, its over-abundance, and its broad dissemination over the Internet, some studies have reported the importance of adapting content—including cultural and linguistic adaptation—to specific sectors of economic activity (11, 42, 56, 58–62). Although commendable in itself, the idea of cultural adaptation is rarely developed and often remains little more than wishful thinking to show sensitivity to the issue, but without providing clear guidance that could be a step toward intercultural competence (23, 63). For instance, in a population study of highly precarious foreign workers in Thailand working in essential services, it was recommended that these workers be encouraged to participate and that some of them become relay persons who could understand the community’s concerns, adapt their actions accordingly, and provide an appropriate response (55).

Faced with varied modes of communication and multiple accesses to information, the neologism “infodemic” has been proposed by some authors (11, 12, 42). A blend of parts of the words “information” and “epidemic,” “infodemic” suggests the idea of large-scale transmission of more or less reliable information, possibly erroneous or rendered obsolete by the rapid progress of science. Infodemics can work in two opposite ways: (1) information is abundant but too dispersed or ill-adapted to the target groups, excessive, confusing, or even misleading; and (2) information is deficient. Quite conceivably, the result may be the same in both situations as communication suffers from a lack of coherence and consistency (57, 64).

The solutions to this problem are not easy to identify in the context of the mass media, which are continually growing. However, the work of Einwiller et al. in Australia, carried out on a sample of 1,033 workers, is instructive as it shows the correlation between the sharing of factual and substantial information on COVID-19, the positive appreciation of communication in the workplace, and the acceptance of managerial decisions on preventive measures to mitigate the risk of SRAS-CoV-2 infection (64). However, very few studies have raised the very contextual problem (and challenge) of constructing an effective communication plan in workplaces when knowledge about SARS-CoV-2 is constantly evolving, leading to multiple changes in health guidance. These challenges are discussed as if the basic information is undisputable and unchanging. Yet the challenge of building a public health or corporate communication plan in a context where the knowledge to be transferred is being generated simultaneously must be addressed, or at the very least, should be part of the message (11).

When evidence and information about a new virus are scarce or likely to change rapidly, it can be difficult to implement control measures. As was the case with SARS-CoV-2 in the early stages of the pandemic, employers and trade unions alike struggled to find the most accurate information (modes of transmission, contagion, hygiene and prevention measures, return to work measures, etc.). And securing up-to-date information did not seem obvious to them, even though public and occupational health services were active in producing and disseminating information. In addition, the development of multilingual information adapted to different sectors took time and not all citizens necessarily knew where to look for it. In this context, basic OHS principles could be mobilized in workplaces, such as the hierarchy of workplace control measures developed by NIOSH to show that design, elimination, and engineering controls should be used first, as they are the most effective when available or feasible (65), and adapted to COVID a few months after the pandemic. The underlying philosophy is that it is always best to try to eliminate hazards first, if possible. If not possible, the first step should be to control the hazard at source, then to isolate people from the hazard, to change the way people work through administrative controls such as policies, training, and providing information in languages that workers understand (66), and finally to provide and ensure use of PPE. These are the universal precautions strategies used to prevent occupational injuries and illnesses, including the transmission of infectious diseases. The ILO has also provided some guidelines on prevention for health workers and responders during public health emergencies, as well as key principles for risk communication with health and other emergency workers during an outbreak (67). The evolving nature of the pandemic should also be introduced as an element of the message, as current recommendations may change and become outdated (66). Providing details of the development of the original and updated material may also be important to dispel any doubts about its accuracy. In addition, language policies and laws that require the use of exclusive languages during a health or public health emergency should be relaxed to allow as many people as possible to understand the information.

The primary objective of our study was to examine the conditions for prevention in essential services where there are precarious working conditions and workers in vulnerable situations. Without asking our informants explicitly about the latter, communication issues emerged as a key theme in our data.

Since the dawn of time, communication has always been at the heart of the human experience. Whether verbal or non-verbal, written, visual or otherwise, any experience of interaction is necessarily a communication experience, which remains a culturally imbedded one (68). However, communication and the exchange or sharing of information takes on its full meaning in the concrete context of interactions. Moreover, it can be said that the modalities of exchange take shape within the very structure of social relations and symbolic capital (69). How do communication challenges affect precarious workers in particular? For example, do agency workers have access to the same information and training as regular workers (70)? Do managers or team leaders pay the same attention to them? Have steps been taken to ensure that the existing material on OHS prevention is provided to everyone and that the content has been adapted culturally or linguistically (22, 71)? Has COVID widened the gaps in OHS prevention (72)?

The pandemic has revealed existing problems and challenges that needed to be addressed by governments long before the SRAS-CoV-2 outbreak. Providing safe and decent working conditions for all workers, regardless of their employment status, is one of the major issues in OHS, and communication is a fundamental part of the equation encompassing every working condition and social position. Yet some workers on the ground with specific vulnerabilities may face additional hardships that are not acceptable in “normal” circumstances and clearly are a bigger concern in times of a health crisis.

## 5 Strengths and limitations

This article highlights some aspects of communication in the workplace that are vital to preventing COVID-19 outbreaks. It shows that access to information cannot be reduced to material access (availability of information), but requires symbolic access (mastery of the cultural code and language level) as well. Various organizational aspects also emerged such as bureaucratic complexity, transparency and confidentiality, and the impact of poor pre-pandemic working relations on crisis management in the workplace. The latter is important and is an area warranting further research given its importance not only for the fight against SARS-CoV-2, but also for OHS in general. Some aspects of managing a health crisis can also be examined in the light of organizational culture, and in particular OHS culture (e.g., training, prevention policy, sickness absence policy, disability, and return-to-work management). One question that could be explored is how the COVID-19 crisis may have prompted a complete review of general OHS practices in some organizations. This study also reveals the importance of better understanding the issues of communication in complex systems where several partners are involved and where divergent viewpoints can be a source of uncertainty and frustration for the general public. The limitations of this project are that the emerging theme of communication was not fully anticipated, and that the researchers may conceivably have only scratched the surface. Another limitation is the sampling bias and study design, which focused exclusively on precarious/low-wage, public-contact workers, who, by the nature of their tasks, must have minimal language skills in either English or French. This population may not necessarily represent the characteristics of precarious workers—such as low literacy levels—in non-public-contact industries (e.g., food processing plants, clothing industry). Other communication issues may emerge from studies that focus on a population other than those working with the public.

In addition, this study took place during the second wave of the pandemic (characterized by the dominant presence of the Delta variant or B.1.617.2). Therefore, it is likely that the concerns of the workplaces mainly reflect the situation at the time (knowledge about the virus, implementation of new mitigation measures, etc.). It is necessary to consider the temporal variable in this type of study, and for future studies, to favor longitudinal designs to attest to the adaptation of workplaces.

This study was not conducted on a large scale and therefore does not provide a generalizable framework for our analyses. On the other hand, it has enabled us, through its in-depth qualitative approach, to gain a better understanding of workers’ health concerns in the field, as well as the concerns of employers and various other stakeholders, from an interactional-systemic perspective, giving us a better grasp of the nature of the communication issues to be addressed during a health crisis. We believe that it is neither premature nor precipitate to recommend implementing a concerted action plan for the communication of health information. In this respect, the recommendations of OSHA and other international bodies already provide a good basis for ensuring that all workers can understand the guidelines, instructions, and fact sheets on any specific health issue, in a language they can understand. Focusing on priority sectors would also be beneficial for the deployment of prevention teams, and it would be up to each jurisdiction and its local partners to establish the criteria.

## 6 Conclusion

The COVID-19 pandemic is not yet over and we have not yet taken all the distance we need to draw all possible lessons from this experience. However, we do know that it has shaken the most vulnerable parts of society; that despite the best intentions, it was not always easy for workplaces to obtain all the answers to their questions; and that communication and response plans were developed simultaneously with the construction of knowledge about SARS-CoV-2. And finally, the availability, but also the quality, of information is an issue in this age of multimedia where it is possible for anyone to develop and disseminate content. The emergence of infodemics calls for vigilance against misinformation. Insofar as the pandemic hit vulnerable populations or those already facing public health and OHS challenges the hardest, this study reminds us of the need to develop targeted, tailored messages that, while not providing all the answers, maintain dialog in workplaces and transparency.

## Data availability statement

The datasets presented in this article are not readily available because they are restricted to the research team. Requests to access the datasets should be directed to daniel.cote@irsst.qc.ca.

## Ethics statement

The study involving human participants was approved by the University of Waterloo Human Research Ethics Board (Protocol certificate number: 42449). Informed participant consent was obtained verbally and recorded before a telephone or video-conference interview.

## Author contributions

DC: Conceptualization, Data curation, Formal analysis, Funding acquisition, Investigation, Methodology, Project administration, Resources, Supervision, Validation, Visualization, Writing – original draft, Writing – review & editing. EM: Conceptualization, Formal analysis, Methodology, Project administration, Resources, Writing – review & editing. A-TH: Data curation, Formal analysis, Investigation, Project administration, Visualization, Writing – review & editing. AL: Data curation, Formal analysis, Investigation, Writing – review & editing. ML: Writing – review & editing. SaM: Conceptualization, Writing – review & editing. ShM: Conceptualization, Writing – review & editing. JA: Data curation, Formal analysis, Investigation, Writing – review & editing. YJ: Data curation, Formal analysis, Investigation, Writing – review & editing. JD: Writing – review & editing.

## Footnotes

^1^Hopwood P, MacEachen E, Côté D, Meyer S, Majowicz S, Huynh AT, et al. (accepted). Occupational pressures of frontline workers enforcing COVID-19 pandemic measures.

## References

[1] Adu-Oppong AA Agyin-Birikorang E. Communication in the workplace: guidelines for improving effectiveness. Global J Commerce Manag Perspec. (2014) 3:208–13.

[2] Laramée A. Une Définition Opératoire Du Système De Communication Organisationnelle. La Communication Dans Les Organisations. Une Introduction Théorique Et Pragmatique. Québec, Qc: Presses De L'université Du Québec (2000).

[3] Brew FP Cairns DR. Do culture or situational constraints determine choice of direct or indirect styles in intercultural workplace conflicts? Int J Intercult Relat. (2004) 28:331–52. doi: 10.1016/j.ijintrel.2004.09.001

[4] Brislin R. Working with cultural differences: Dealing effectively with diversity in the workplace. Westport, Ct: Praeger Publishers/Greenwood Publishing Group (2008).

[5] Jacques F. Dialogue, dialogism, interlocution. L’orientation scolaire et professionnelle [online]. 29. doi: 10.4000/osp.5866

[6] Werner E. Toward a theory of communication and cooperation for multiagent planning In: Vardi MY, editor. Theoretical aspects of reasoning about knowledge: Proceedings of the second conference. Los Altos, Ca: Morgan Kaufmann Publishers (1988)

[7] Putnam LL Mumby DK. The Sage handbook of organizational communication. US: Sage (2013).

[8] Berger CR. Interpersonal communication: theoretical perspectives, future prospects. J Commun. (2005) 55:415–47. doi: 10.1111/j.1460-2466.2005.tb02680.x

[9] Venuelo C Gelo OCG Salvatore S. Fear, affective semiosis, and management of the pandemic crisis: Covid-19 as semiotic vaccine? Clin Neuropsychiatry. (2020) 17:117–30. doi: 10.36131/CN20200218 34908982 PMC8629038

[10] Magarini FM Pinelli M Sinisi A Ferrari S De Fazio GL Galeazzi GM. Irrational beliefs about Covid-19: a scoping review. Int J Environ Res Public Health. (2021) 18:1–21. doi: 10.3390/ijerph18199839, PMID: 34639241 PMC8508358

[11] Ratzan SC Sommariva S Rauh L. Enhancing Global Health communication during a crisis: lessons from the Covid-19 pandemic. Public Health Res Prac. (2020) 30:E3022010. doi: 10.17061/phrp3022010 32601655

[12] Yoon S Mcclean ST Chawla N Kim JK Koopman J Rosen CC. Working through an "Infodemic": the impact of Covid-19 news consumption on employee uncertainty and work behaviors. J Appl Psychol. (2021) 106:501–17. doi: 10.1037/apl0000913 34014706

[13] Gilson L. Trust and the development of health care as a social institution. Soc Sci Med. (2003) 56:1453–68. doi: 10.1016/S0277-9536(02)00142-9 12614697

[14] Kupferschmidt K. Ending coronavirus lockdowns will be a dangerous process of trial and error. Science. (2020) 369:124–5. doi: 10.1126/science.abc2507, PMID: 32646976

[15] WHO. (2020). Statement On The Second Meeting Of The International Health Regulations (2005) Emergency Committee Regarding The Outbreak Of Novel Coronavirus (2019-Ncov) [Online]. Geneve (Swizerland): World Health Organization. Available at: https://www.who.int/news/item/30-01-2020-statement-on-the-second-meeting-of-the-international-health-regulations-(2005)-emergency-committee-regarding-the-outbreak-of-novel-coronavirus-(2019-ncov) [Accessed].

[16] Cubrich M. On the frontlines: protecting low-wage workers during Covid-19. Psychol Trauma. (2020) 12:S186–7. doi: 10.1037/tra0000721, PMID: 32551757

[17] Greenhalgh T Ozbilgin M Contandriopoulos D. Orthodoxy, Illusio, and playing the scientific game: a Bourdieusian analysis of infection control science in the Covid-19 pandemic [version 3; peer review: 2 approved]. Wellcome Open Res. (2021) 6:126. doi: 10.12688/wellcomeopenres.16855.3, PMID: 34632088 PMC8474098

[18] Randall K Ewing ET Marr LC Jimenez JL Bourouiba L. How did we get Here: what are droplets and aerosols and how far do they go? A historical perspective on the transmission of respiratory infectious diseases. Interface Focus. (2021) 11:20210049. doi: 10.1098/rsfs.2021.0049, PMID: 34956601 PMC8504878

[19] Finset A Bosworth H Butow P Gulbandsen P Hulsman RL Pieterse AH. Editorial: effective health communication – a key factor in fighting the Covid-19 pandemic. Patient Educ Couns. (2020) 103:873–6. doi: 10.1016/j.pec.2020.03.027, PMID: 32336348 PMC7180027

[20] Horton R. Offline: Covid-19 is not a pandemic. Lancet. (2020) 396:874. doi: 10.1016/S0140-6736(20)32000-6, PMID: 32979964 PMC7515561

[21] Singer M Rylko-Bauer B. The Syndemics and structural violence of the Covid pandemic: anthropological insights on a crisis. Open Anthropol Res. (2021) 1:7–32. doi: 10.1515/opan-2020-0100

[22] Benach J Pericàs JM Martínez-Herrera E Bolíbar M. Public health and inequities under capitalism: systemic effects and human rights In: Vallverdú J Puyol A Estany A, editors. Philosophical and methodological debates in public health. Cham, Switzerland: Springer (2019)

[23] Côté D Durant S Maceachen E Majowicz S Meyer S Huynh A-T. A rapid scoping review of Covid-19 and vulnerable workers: intersecting occupational and public health issues. Am J Ind Med. (2021) 64:551–66. doi: 10.1002/ajim.23256, PMID: 34003502 PMC8212119

[24] Díaz Bretones F Santos A. Health, safety and well-being of migrant workers: New hazards. New Workers, London, Uk: Springer (2020).

[25] Flynn MA Check P Steege AL Siven JM Syron LN. Health equity and a paradigm shift in occupational safety and health. Int J Environ Res Public Health. (2021) 19, 1–13. doi: 10.3390/ijerph19010349, PMID: 35010608 PMC8744812

[26] Kapadia F. Public health practice and health equity for vulnerable workers: a public health of consequence, may 2023. Am J Public Health. (2023) 113:480–1. doi: 10.2105/AJPH.2023.307268, PMID: 37018693 PMC10088941

[27] Gravlee CC. Systemic racism, chronic health inequities, and Covid-19: a Syndemic in the making? Am J Hum Biol. (2020) E23482:1–8. doi: 10.1002/ajhb.23482 PMC7441277 32754945

[28] Katikireddi SV Lal S Carrol ED Niedzwiedz CL Khunti K Dundas R. Unequal impact of the Covid-19 crisis on minority ethnic groups: a framework for understanding and addressing inequalities. J Epidemiol Community Health. (2021) 75:970–4. doi: 10.1136/jech-2020-216061, PMID: 33883198 PMC8458062

[29] Sachs JD Karim SSA Aknin L Allen J Brosbøl K Colombo F. The lancet commission on lessons for the future from the Covid-19 pandemic. Lancet. (2022) 400:1224–80. doi: 10.1016/S0140-6736(22)01585-9 36115368 PMC9539542

[30] Lippel K Thébaud-Mony A. Precarious employment and the regulation of occupational health and safety: prevention, compensation and return to work In: Sheldon P Gregson S Landsbury RD Sanders K, editors. The regulation and management of workplace health and safety. New York: Routledge (2021)

[31] Mcclure ES Vasudevan P Bailey Z Patel S Robinson WR. Racial capitalism within public health: how occupational settings drive Covid-19 disparities. Am J Epidemiol. (2020) 189:1244–53. doi: 10.1093/aje/kwaa126, PMID: 32619007 PMC7337680

[32] Berdahl TA. Racial/ethnic and gender differences in individual workplace injury risk trajectories: 1988-1998. Am J Public Health. (2008) 98:2258–63. doi: 10.2105/AJPH.2006.103135, PMID: 18235072 PMC2636522

[33] Cranford CJ Vosko LF Zukewich N. The gender of precarious employment in Canada. Relations Indus / Indus Relations. (2003) 58:454–82. doi: 10.7202/007495ar

[34] Underhill E Quinlan M. How precarious employment affects health and safety at work: the case of temporary agency workers. Relations Indus / Indus Relations. (2011) 66:397–421. doi: 10.7202/1006345ar

[35] Kalleberg AL Vallas SP. Probing precarious work: theory, research, and politics. Res Sociol Work. (2018) 31:1–30. doi: 10.1108/S0277-283320170000031017

[36] Kreshpaj B Orellana C Burström B Davis L Hemmingsson T Johansson G. What is precarious employment? A systematic review of definition and Operationalizations from quantitative and qualitative studies. Scand J Work Environ Health. (2020) 46:235–47. doi: 10.5271/sjweh.3875 31901944

[37] Fortin M-F Gagnon J. Fondements Et Étapes Du Processus De Recherche. Méthodes Quantitatives Et Qualitatives. 3rd ed. Montréal, Qc: Chenelière Éducation (2015).

[38] Wilson AD Onwuegbuzie AJ Manning LP. Using paired depth interviews to collect qualitative data. Qual. Rep. (2016) 21:1549–1573. doi: 10.46743/2160-3715/2016.2166

[39] Miles MB Huberman AM Saldana J. Qualitative data analysis: A methods sourcebook. Fourth ed. Los Angeles, London, New Delhi, Singapore, Washington Dc: Sage Publications (2018).

[40] Clarke AE. Situational analyses: grounded theory mapping after the postmodern turn. Symb Interact. (2003) 26:553–76. doi: 10.1525/si.2003.26.4.553

[41] Basch CH Mohlman J Hillyer GC Garcia P. Public health communication in time of crisis: readability of on-line Covid-19 information. Disaster Med Public Health Prep. (2020) 14:635–7. doi: 10.1017/dmp.2020.151, PMID: 32389144 PMC7235310

[42] Casalegno C Civera C Cortese D. Covid-19 in Italy and issues in the communication of politics: bridging the knowledge-behaviour gap. Knowledge Manag Res Prac. (2021):459–467. doi: 10.1080/14778238.2020.1860664

[43] Malecki KMC Keating JA Safdar N. Crisis communication and public perception of Covid-19 risk in the era of social media. Clin Infect Dis. (2020) 72:697–702. doi: 10.1093/cid/ciaa758 PMC7337650 32544242

[44] Maceachen E Clarke J Franche R-L Irvin E. Systematic review of the qualitative literature on return to work after injury. Scand J Work Environ Health. (2006) 32:257–69. doi: 10.5271/sjweh.1009, PMID: 16932823

[45] Pransky G Borkan JM Young AE Cherkin DC. Are we making Progress? The tenth international forum for primary care research on low Back pain. Spine. (2011) 36:1608–14. doi: 10.1097/BRS.0b013e3181f6114e 21245787

[46] Benach J Muntaner C Santana V. Employment conditions and health inequalities final report to the who commission on social determinants of health (Csdh). Barcelone (Espagne): Icaria editorial (2007).

[47] Côté D Dubé J Gravel S Gratton D White BW. Cumulative stigma among injured immigrant workers: a qualitative exploratory study in Montreal (Quebec, Canada). Disabil Rehabil. (2020) 42:1153–66. doi: 10.1080/09638288.2018.1517281 30686038

[48] Crollard A De Castro AB Tsai JH. Occupational trajectories and immigrant worker health. Workplace Health Safety. (2012) 60:497–502. doi: 10.1177/216507991206001105 23092177 PMC3654177

[49] Lay AM Kosny A Aery A Flecker K Smith PM. The occupational health and safety vulnerability of recent immigrants accessing settlement services. Can J Public Health. (2018) 109:303–11. doi: 10.17269/s41997-018-0063-4, PMID: 29981078 PMC6964557

[50] Sherpa. Covid-19, migration et Diversité. Montréal, Qc: Sherpa University Institute (2021).

[51] Québec. Coronavirus, Covid-19, multilingual tools. Montréal, Qc: Santé Montréal (2021).

[52] Cnesst. Covid-19 Toolkit. Québec, Qc: Commission Des Normes, De L'équité, De La Santé Et De La Sécurité Du Travail (2021).

[53] Ontario. (2021). Covid-19 communication resources. Find resources in multiple languages to help local communication efforts in responding to Covid-19. Toronto, On: Government Of Ontario. Available at: https://www.ontario.ca/page/covid-19-communication-resources [Accessed].

[54] Lee J Kim M. Estimation of the number of working population at high-risk of Covid-19 infection in Korea. Epidemiol Health. (2020) 42:E2020051. doi: 10.4178/epih.e2020051 32660216 PMC7871163

[55] Rojanaworarit C El Bouzaidi S. Building a resilient public health system for international migrant workers: a case study and policy brief for Covid-19 and beyond. J Health Res. (2021) 36:898–907. doi: 10.1108/JHR-01-2021-0035

[56] Wild A. Kunstler B. Goodwin D. Skouteris H. Zhang L. Kufi M.. (2020). Communicating Covid-19 health information to culturally and linguistically diverse (Cald) communities: the importance of partnership, co-design, and Behavioural and implementation science. Public Health Res Pract. (2021) 31:1–5. doi: 10.17061/phrp3112105 33690789

[57] Poonia SK Rajasekaran K. Information overload: a method to share updates among frontline staff during the Covid-19 pandemic. Otolaryngol--Head Neck Surg: Official J American Acad Otolaryngol Head Neck Surg. (2020) 163:60–2. doi: 10.1177/0194599820922988 32315261

[58] Bui DP Mccaffrey K Friedrichs M Lacross N Lewis NM Sage K. Racial and ethnic disparities among Covid-19 cases in workplace outbreaks by industry sector - Utah, march 6-June 5. MMWR Morb Mortal Wkly Rep. (2020) 69:1133–8. doi: 10.15585/mmwr.mm6933e3, PMID: 32817604 PMC7439983

[59] Moore JT Ricaldi JN Rose CE Fuld J Parise M Kang GJ. Disparities in incidence of COVID-19 among underrepresented racial/ethnic groups in counties identified as hotspots during June 5–18, 2020 — 22 states, February–June 2020. MMWR Morb Mortal Wkly Rep. (2020) 69:1122–6. doi: 10.15585/mmwr.mm6933e1, PMID: 32817602 PMC7439982

[60] Pouliakas K Branka J. Eu jobs at highest risk of Covid-19 social distancing: Will the pandemic exacerbate the labour market divide? Luxembourg: Publications Office Of The European Union (2020).

[61] Smith C. The structural vulnerability of healthcare workers during Covid-19: observations on the social context of risk and the equitable distribution of resources. Soc Sci Med. (2020) 258:113119. doi: 10.1016/j.socscimed.2020.113119, PMID: 32534301 PMC7280115

[62] Sterling MR Tseng E Poon A Cho J Avgar AC Kern LM. Experiences of home health care workers in new York City during the coronavirus disease 2019 pandemic: a qualitative analysis. JAMA Intern Med. (2020) 180:1453–9. doi: 10.1001/jamainternmed.2020.3930, PMID: 32749450 PMC7404061

[63] Côté D Dubé J Gravel S. Developing intercultural competence in a complex organizational structure: a case study within Quebec’s workers’ compensation board. J Appl Rehabil Couns. (2022) 53:170–92. doi: 10.1891/JARC-D-21-00004

[64] Einwiller S Ruppel C Stranzl J. Achieving employee support during the Covid-19 pandemic – the role of relational and informational crisis communication in Austrian organizations. J Commun Manag. (2021) 25:233–55. doi: 10.1108/JCOM-10-2020-0107

[65] Zontek TL Ogle BR. Introduction to industrial hygiene. In: MA Friend JP Kohn, editor. Fundamentals of occupational safety and health, 8th edition. Lanham, ML: Bernan Press (2023). 97–130.

[66] OSHA. Control and prevention: interim guidance for job task associated with increased risk of exposure to SARS-CoV-2. occupational safety and health administration, U.S. Department of Labor (2023). Availale at: https://www.osha.gov/coronavirus/control-prevention.

[67] ILO. Occupational safety and health in public health emergencies: A manual for protecting health workers and responders. Geneva, Switzerland: World Health Organization and International Labour Organization (2018). Available at: https://www.who.int/publications/i/item/9789241514347

[68] Côté D. Intercultural communication in health care: challenges and solutions in work rehabilitation practices and training: a comprehensive review. Disabil Rehabil. (2013) 35:153–63. doi: 10.3109/09638288.2012.687034 22616895

[69] Bourdieu P. Espace Social Et Pouvoir Symbolique. Choses Dites. Paris: Minuit (1987).

[70] Dubé J Gravel S. Preventive practices for workers from personnel placement agencies in permanent or temporary positions: comparison between immigrant and non-immigrant workers (in French: les Pratiques Préventives Auprès des Travailleurs D’agences De location De personnel Temporaire Ou permanent: Comparaison entre les Travailleurs immigrants et non immigrants). Pistes. (2014) 16:1–18. doi: 10.4000/pistes.3911

[71] Falicov C Niño A D'urso MS. Expanding possibilities: Flexibility and solidarity with under resourced immigrant families during the Covid-19 pandemic. Fam Process. (2020) 59:865–82. doi: 10.1111/famp.12578 32663315 PMC7405176

[72] Krouse HJ. Covid-19 and the widening gap in health inequity. Otolaryngol Head Neck Surg. (2020) 163:65–6. doi: 10.1177/0194599820926463, PMID: 32366172

